# Developmental paths of the associations between visuospatial working memory and numerical processing

**DOI:** 10.1007/s00426-026-02240-6

**Published:** 2026-02-07

**Authors:** Sarit Ashkenazi, Anna Adi

**Affiliations:** Learning Disabilities, The Hebrew University of Jerusalem, The Seymour Fox School of Education, Jerusalem, Israel

**Keywords:** Symbolic quantity representations, Non-symbolic quantity representations, Development, Spatial short-term memory, Spatial working memory

## Abstract

The present study examines the role of spatial abilities in numerical processing in children using an application administered on parents’ smartphones. The study comprised four tasks: (1) spatial short-term memory (2) spatial working memory (3) non-symbolic comparison, and (4) symbolic comparison. A total of 541 children performed all four tasks (mean age = 6.41, SD = 4.05), and a conjunction analysis was conducted on the data collected from all four tasks. One of the main goals of the present study was to understand the role of spatial abilities in numerical processing and the modulating effect of age on the relationship between them. We found that quantity comparison tasks are directly associated, primarily, with spatial short-term memory and also (but to a lesser degree) with spatial working memory. These two associations decrease with age. We suggest that younger children tend to use a spatial strategy during numerical comparison tasks. However, when symbolic and non-symbolic comparisons were combined into a unified model, no direct links were found between symbolic comparison and spatial abilities. Furthermore, in the unified model, age affected the non-symbolic comparison abilities, but not the symbolic comparison abilities. These results suggest that in young children, there is no direct link between symbolic numerical representations and spatial abilities. Accordingly, these results uniquely demonstrate that symbolic representation is built upon non-symbolic representation.

**Supplementary information:**

The online version contains supplementary material available at 10.1007/s00426-026-02240-6.

## Introduction

Symbolic arithmetic is a relatively recent cultural invention. However, humans have an intuitive ability to manipulate and understand approximate magnitudes, which exists primarily and independently of training and culture (Feigenson & Halberda, 2004; Halberda & Feigenson, 2008; Halberda et al., 2008, 2012). Evidence from infants, preschool children, and adults, as well as from non-human primates, has consistently shown that processing and manipulation of quantity are preverbal abilities based on defined neurological circuits (Ashkenazi et al., 2012; Dehaene et al., 1999, 2003; Feigenson & Halberda, 2004; Halberda & Feigenson, 2008). This preverbal quantity representation is spatial in nature (Ashkenazi et al., 2013).

Spatial abilities are some of the cognitive building blocks of mathematical abilities (Cornu et al., 2018; Geer et al., 2019; Lauer & Lourenco, 2016). Spatial ability is the capacity to understand, reason, and remember the visual and spatial relations between objects (Pellegrino, Alderton, & Shute., 1984). A few cognitive and brain mechanisms create the links between spatial and basic numerical abilities, and later, also with mathematical performance. First, there is a common genetic influence on mathematical and spatial abilities (Tosto et al., 2014). Second, similar brain regions, including the intraparietal sulcus (IPS) and other regions in the frontoparietal network, are associated with numerical processing and spatial processing (Hubbard et al., 2005; Rotzer et al., 2009).

In addition, from very early stages, infants tend to connect quantities and space (de Hevia, 2016; de Hevia & Spelke, 2010). A recent study, performed in our laboratory, tested the role of domain-general factors (spatial abilities, executive function, working memory, attention, and fine motor skills) in numerical processing in young children (*n* = 339) of various ages (between 3 and 8 years). We found that spatial skills directly and strongly affect numerical skills, and specifically, spatial working memory (WM) was the strongest predictor of all the spatial abilities evaluated (Ashkenazi & Adi, 2023).

The development of mathematical skills heavily relies on WM, making it one of the most extensively studied cognitive abilities underlying mathematical performances (Alloway & Alloway, 2010; Bull et al., 2008; Bull & Lee, 2014; Cragg & Gilmore, 2014; De Smedt et al., 2009; DeStefano & LeFevre, 2004; Friso-van den Bos et al., 2013; Passolunghi & Costa, 2019; Peng et al., 2012; Raghubar et al., 2010). However, WM is not a unitary system. The classic working memory model of Baddeley and Hitch (1974) includes the central executive component that manipulates information, and two specialized systems: a phonological loop and a visuospatial sketchpad. The phonological loop is responsible for the short-term storage of verbal information, while the visuospatial sketchpad handles visuospatial information. However, more current approaches to WM have found evidence of modal specificity within the working memory, suggesting a dissociation between the ability to manipulate visuospatial and verbal information (Saults & Cowan, 2007). This approach differentiates between components of short-term storage and WM of visuospatial and verbal information. WM, by this approach, requires additional processing and is also comprised of verbal and visuospatial components (Szűcs, 2016). However, the longitudinal involvement of different components of WM in numerical and arithmetical processing in different stages of development is still debated.

### WM components and mathematics

Each WM component can play an important and unique role in mathematics performance (DeStefano & LeFevre, 2004; Friso-van den Bos et al., 2013; Szűcs, 2016). In addition, the relationship between WM components and mathematics changes throughout development (Caviola et al., 2020). In the initial stages of acquiring quantity understanding in preschoolers (ages between 4.5 and 6 years old), before the acquisition of verbal exact numerical representation, spatial WM plays a significant role, due to the usage of a mental model (Rasmussen & Bisanz, 2005). However, later (ages between 6.2 and 7.7 years old), verbal WM becomes more significant. In a longitudinal study, Bull et al. (2008) found that verbal WM (tested in age 4.6 years, approximately) predicted mathematics scores in first grade (7-year-olds), while spatial WM (tested in age 4.6 years, approximately) uniquely predicted them in third grade (9-year-olds). Cross-sectional studies have found a similar pattern (Alloway & Passolunghi, 2011; Meyer et al., 2010). Li and Geary (2013) found that improvement in spatial WM, measured from first (approximately 7 years old) to fifth grade (approximately 9 years old), predicted mathematics scores at the end of fifth grade. They also discovered that this correlation increases with age, as the mathematical material becomes more complex.

To conclude, the contribution of spatial short-term memory and WM to number processing appears to shift across development. In preschoolers, spatial short-term memory and WM show strong associations with numerical processing, likely because children rely on spatial mental models to represent quantities before mastering exact symbolic representations (Rasmussen & Bisanz, 2005). In the early elementary years, as verbal numerical representations emerge, the influence of spatial short-term memory and WM becomes less pronounced (Meyer et al., 2010). From around third grade (approximately 9 years of age) onward, however, spatial short-term memory, and particularly spatial WM, again emerge as unique predictors of numerical abilities, especially in more complex and less automatic numerical tasks (Li & Geary, 2017).

Although a few studies have examined the developmental shift in the role of visuospatial short-term and WM in numerical and mathematical processing (e.g., Rasmussen & Bisanz, 2005; Meyer et al., 2010), their findings are inconsistent because they were based on varying mathematical and spatial tasks. Moreover, a few aspects of the link between spatial skills and number processing have yet to be explored, in particular, the effect of development on these associations.

### The present study

Numerical and mathematical abilities are closely related, and spatial abilities are composed of multiple components (Tosto et al., 2014). However, it has been suggested that visuospatial working memory constitutes an important domain-general source of vulnerability in the development of arithmetic cognition (Ashkenazi et al., 2013), and that it is the strongest predictor of numerical abilities in children (Ashkenazi & Adi, 2023). Accordingly, the present study focuses on the specific role of visuospatial working memory in mathematical abilities, while omitting other aspects of spatial abilities.

The main objective of this study is to examine the changes that occur in the relationship between numerical and spatial abilities throughout the different stages of development, by testing the same numerical and spatial tasks in a wide age range (2.5–6.5 years) and on a large sample of children. We tested symbolic and non-symbolic quantities comparison as an early marker of numerical abilities. We believe that the intensity of the associations between spatial short-term memory, WM, and numerical abilities will decrease with age (e.g., Rasmussen & Bisanz, 2005; Meyer et al., 2010), for two reasons. First, in this study, we used symbolic and non-symbolic numerical comparisons, relatively simple tasks for school age children. Because relatively simple tasks require less spatial processing (Li & Geary, 2017), we expect stronger associations in younger children compared to older children. Second, with age, the use of a mental model, which is based on spatial representation (Rasmussen & Bisanz, 2005), decreases due to increasing verbal abilities.

The second goal of the present study is to look at the differential role of spatial short-term memory and WM in symbolic and non-symbolic numerical representations throughout development. Two popular theories explain the relationship between symbolic and non-symbolic numerical representation. The first theory suggests that humans have two independent systems for numerical representation: a symbolic exact numerical representation system and a non-symbolic approximate numerical system (Noel & Rousselle, 2011). The second theory suggests that the symbolic numerical system is built upon a non-symbolic numerical system (Halberda & Feigenson, 2008; Halberda et al., 2008). Understanding the role of spatial abilities in numerical representation can provide insight into the relationship between symbolic and non-symbolic representations during development. Due to the spatial component involved in non-symbolic numerical representations, stronger associations between spatial short-term memory and WM to non-symbolic representations are expected, compared to symbolic representations.

According to the first theory, which suggests two independent systems for numerical representation, one can expect independent relations between short-term memory and WM to non-symbolic and symbolic comparisons. However, according to the second theory, which suggests that symbolic processing is built upon non-symbolic processing, one can only expect a direct link between short-term memory and WM to non-symbolic comparisons.

However, it is still unclear whether there will be any independent association between symbolic numerical representations and spatial abilities. This independent association can only be found if the symbolic numerical system is partially or fully autonomous and not only directly mapped upon the non-symbolic representation.

To achieve these goals in the current study, a very large sample of children of various ages performed (1) a symbolic comparison task, (2) a non-symbolic comparison task, (3) the Corsi Blocks forward task, and (4) the Corsi Blocks backwards task. All tasks were performed using the GiantLeap app. Some of the data from the current study were already used in former manuscripts, including symbolic and non-symbolic comparison and the Corsi result, but with different analyses (Ashkenazi, 2024; Ashkenazi & Adi, 2023).

## Materials and methods

### Participants

Most of the participants were from the United States (80%), and others were native English speakers from other countries (such as Australia, Canada and the United Kingdom). All participants performed the tasks using the GiantLeap app. Participants were recruited through a newspaper advertisement and online articles describing the GiantLeap app, both of which contained a participation link. They did not receive any compensation for participation other than feedback about their relative scores.

Our dataset of children who completed a minimum of 1 task from the application included 4,127 children, who were evaluated between 2020 and 2021. 65% of the children were boys. Only a small portion of these children were included in the current analysis, according to the following criteria. 1) Children between the ages of 2.5 to 7.5 years. 541 children completed all four tasks (mean age = 6.41, S.D. = 4.05), and a conjunction analysis was performed on data gathered from all four tasks (a listwise deletion procedure on missing cases was used). The dropout rates were high due to the inability to complete the series of tests. Specifically, the Corsi block tapping task was very hard for 3-year-olds, and only 14% of the children could complete the task (especially backward). We also excluded participants in the age group of 6.50 to 7.50 due to a small sample size compared to the other groups (*n* = 30). Table 1 presents the number of children in each age group that completed the Corsi backward task to show the large dropout rates that are also age-dependent.

**Table 1:** Number of children by age group that completed (the first column) or did not complete (the second column) the corsi backward task. The participants in the did not complete column represent participants who started the task but did not finish it

Age group	Completed	Did not complete	Total
3	73 (14%)	438 (86%)	511
4	218 (29%)	522 (71%)	740
5	199 (42%)	271 (58%)	470
6	155 (49%)	162 (51%)	317

Percentage in parentheses

### Tools

The children used their parents’ smartphones to access the GiantLeap app, a child development evaluation tool designed for use in non-controlled environments. The evaluation process is divided into two modules: (a) a series of engaging tasks for the child, and (b) questionnaires for the parents. In the current study, we analyzed only a subset of four tasks: symbolic and non-symbolic comparisons, and the Corsi forward and backward. The full results are reported in other studies (e.g., Ashkenazi & Adi, 2023).

The child task module includes measures of attention (continuous performance task), numerical processing (symbolic comparison, non-symbolic comparison, and enumeration), spatial abilities (mental rotation), spatial working memory (Corsi forward and backward), and executive function (color–shape task). All tasks were designed as child-friendly games.

In addition, the GiantLeap app includes several parent questionnaires assessing the child’s self-esteem, attention, gross motor skills, fine motor skills, and language abilities, as well as an emotional screener (emotional symptoms, hyperactivity, peer problems, conduct problems, and prosocial abilities). Tasks were completed over several sessions. Before the beginning of each task, children received verbal instructions (e.g., “Let’s call mom or dad so we can learn how to play the next game together”). After completing each task, children were asked to rate the activity and received a puzzle piece as a reward.

**Fig. 1:**
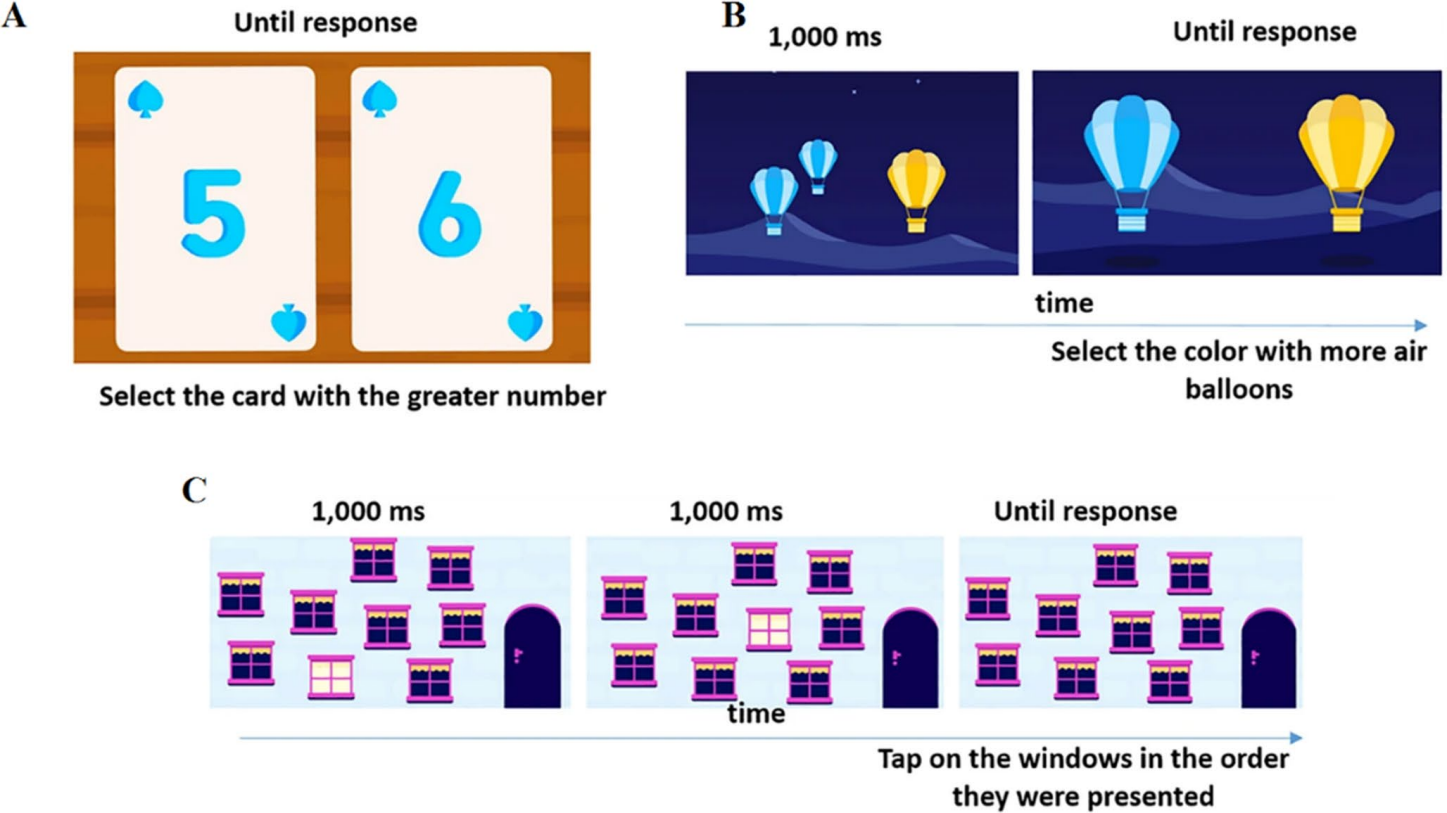
Example screen shots of the tasks used for this study as displayed on the app. **A** Symbolic comparison, **B** Non-symbolic comparison, **C** Corsi blocks forward task, (for backwards Corsi blocks task, same imagery but with different instructions)

Further details about the tasks are provided below, and their structure is illustrated in Fig. 1. For tasks adopted from other studies, some adjustments were made to increase child-friendliness, as described below.

### Symbolic comparison

Two white cards with a big blue number in the middle of each were presented on a brown wooden table-like background. The cards contained different numbers (between 2 and 6). The child was asked to tap with his finger on the card with the higher value number. The cards were presented until response, followed by a new trial. The task started with a demonstration and a verbal instruction, followed by 6 practice trials with feedback. Then the child went on to perform the experiment that contained 16 trials: 8 trials with close number distance (distance 1: the pairs 2–3, 5–6, 3 − 2, 6 − 5, every pair was presented twice), and 8 trials with far number distance (distance 3: 2–5, 3–6, 5 − 2, 6 − 3, every pair was presented twice).

The dependent measure was an efficiency score, calculated as follows: (1) mean reaction times and accuracy rates were transformed into z-scores; (2) reaction time z-scores were multiplied by − 1 to obtain inverted scores; and (3) the inverted reaction time scores and standardized accuracy scores were averaged to yield a single efficiency measure (Ashkenazi and Silverman., 2017).

### Non-symbolic comparison

Images of two groups of hot air balloons (blue and yellow) were shown for 1000 milliseconds, followed by images of two single hot air balloons, a blue one and a yellow one. The child was verbally asked to tap the hot air balloon with the color corresponding to the larger group of balloons showed previously. The task started with a demonstration and verbal instruction, followed by 6 practice trials with feedback. The child then went on to conduct 16 trials, varying the ratio. The specific pairs were: (the first number represents yellow, and the second number represents blue) 1–2; 2–3; 2–4; 4–5; 5–6 and the opposite; 6–7 and the opposite; 7–8 × 2; 8–9 × 2 and the opposite; 9–10 × 2 and the opposite. In half of the trials, the total area of the two sets of air balloons was identical, and in the other half, the average size of the objects in the two sets of air balloons was identical. The task was adopted from Halberda and Feigenson (2008); Halberda et al. (2008). Compared to the original version of the task, we used air balloons instead of simple dots and reduced the number of repetitions per condition. The dependent measure was an efficiency score, calculated as follows: (1) mean reaction times and accuracy rates were transformed into z-scores; (2) reaction time z-scores were multiplied by − 1 to obtain inverted scores; and (3) the inverted reaction time scores and standardized accuracy scores were averaged to yield a single efficiency measure (Ashkenazi and Silverman., 2017).

### Corsi blocks forward (testing spatial short-term memory) (adopted form Berch et al., 1998).

The task started with a cover story, “Danny lives in a house with many windows. The windows light up one after the other. Help Danny remember the order in which the windows light up. Then, tap on the windows in that order, and tap on the door when you are done”.

The child was presented with 9 windows scattered on the screen during the task. On each trial, a subset of the windows lit up in a pseudo-random order (each one for 1,000ms), and the child was then asked to tap on the windows in the same order (first to last). The task started with a demonstration and verbal instructions, followed by 6 practice trials with sequence lengths varying between 2 and 4 windows, with feedback. Then the child continued with the experiment for a minimum of 3 trials, up to a maximum of 21 trials, according to individual accuracy. Each sequence length (2 windows, 3 windows, etc.) consisted of 3 trials. If the child succeeded in 2 out of 3 trials in a given length, the sequence length increased by 1, and the task continued; otherwise, the task ended. The first length was 2 windows, and the longest possible sequence was 7 windows long. The score of the participant was the span, the maximum length that an individual participant could correctly recall. Compared to the original version of the task, we used windows instead of squares. The depended measurement was span, the maximum number of squares that were correctly remembered.

### Corsi blocks backwards (testing spatial WM) (adopted form Berch et al., 1998).

All the conditions were the same as in the Corsi blocks task (see “Corsi blocks forward (testing spatial short-term memory)" section) except for the instruction: “Tap on the windows in the reverse order and tap on the door when you are done.”

The score of the participant was the span, the maximum length that an individual participant could correctly recall. Compared to the original version of the task, we used windows instead of squares. The depended measurement was span, the maximum number of squares that were correctly remembered.

### Analysis

In the first step, we examined developmental effects on visuospatial short-term memory and WM by comparing different age groups of children. Next, using multiple moderation analyses with age treated as a continuous variable, we tested whether age moderated the relationship between these memory abilities and performance on symbolic and non-symbolic comparison tasks. We then performed a path analysis to evaluate the combined effects of all the variables. For the symbolic and non-symbolic comparison tasks, performance was quantified using an efficiency score. We calculated this score by first standardizing (z-scoring) reaction time and accuracy, then multiplying the reaction time z-score by − 1 to invert it (so that higher values indicate better performance), and finally averaging this inverted reaction time z-score with the accuracy z-score (Ashkenazi and Silverman., 2017). For the Corsi forward and backward tasks, we used the span length as the performance measure. Table 2 presents the descriptive statistics for each age group on each task.

**Table 2:** Descriptive statistics by task and age group for all tasks used in the current Study. The values without parentheses represent accuracy rates (in the symbolic and the non symbolic tasks), and span (in the corsi tasks). The values in the parentheses represent reaction times

		Symbolic accuracy (RT in sec)			Non Symbolic accuracy (RT in sec)			Corsi forward Span (S.D)			Corsi backward Span (S.D)		
Age Group	N	M	Min	Max	M	Min	Max	M	Min	Max	M	Min	Max
3	40	0.94 (0.56)	0.50 (0.12)	1 (10.53)	0.66 (0.42)	0.44 (0.15)	0.94 (3.73)	3.25 (1.42)	1.67	6.67	3.14 (1.27)	1.67	6.67
4	149	0.94 (0.58)	0.38 (0.12)	1 (13.39)	0.68 (0.40)	0.50 (0.12)	1 (10.00)	3.16 (0.92)	1.67	6.00	2.78 (0.95)	1.67	6.00
5	133	0.96 (0.56)	0.63 (0.07)	1 (5.76)	0.72 (0.29)	0.312 (0.13)	1 (20.90)	3.46 (0.71)	1.67	5.67	3.05 (0.86)	0.33	6.67
6	89	0.98 (0.68)	0.63 (0.06)	1 (2.13)	0.76 (0.43)	0.50 (0.12)	1 (1.80)	3.91 (0.86)	2.00	7.00	3.43 (0.87)	1.67	5.67

*M* mean, *S.D* standard deviation

## Results

### Development of Spatial span

**Fig. 2:**
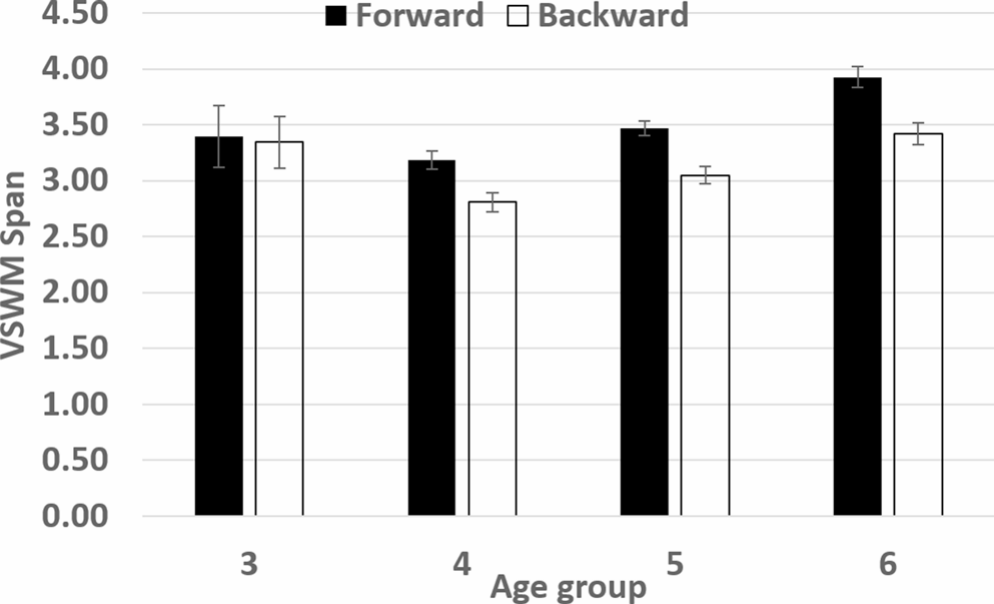
Span as a function of age group and task (corsi forward, corsi backward)

To examine how visuospatial short-term and WM change throughout development, we first grouped the children by age. Age group 3 included children between 2.5 and 3.5 years old; age group 4 included children older than 3.5 and up to 4.5 years old; and so on, up to age group 6. We then conducted a mixed ANOVA with direction (forward vs. backward) as a within-subjects factor and age group (3–6) as a between-subjects factor. This analysis revealed a main effect of direction *F*(1, 374) = 31.55, *p* =.01, partial η² = 0.08, a main effect of age group, *F* (3, 374) = 13.10, *p* =.01, partial η² = 0.01, with no direction x age group interaction *F*(3, 374) = 1.64, *p* =.18, partial η² = 0.01. As shown in Fig. 2, children performed better in forward direction (M = 3.46, S.D.= 0.96) than in backward direction (M = 3.07, S.D.= 0.98). Additionally, except for age group 3 and 4, children performed better over time (M age 3 = 3.37, S.D.= 1.44; M age 4 = 3.00, S.D.= 0.96; M age 5 = 3.26, S.D.= 0.77; M age 6 = 3.67, S.D.= 0.88) (see Fig. 2). It is important to note that the tasks were very difficult for three-year-old children. Consequently, the performance of those who managed to complete the tasks cannot be taken as representative of the whole group.

### Relationship between different domains - the effect of age

We also investigated whether age affects the relationship between visuospatial short-term memory and WM and symbolic and non-symbolic magnitude comparisons. Age served as a continuous variable. First, we used moderation analysis (model 4, one moderation in the PROCESS software) using the PROCESS macro (Hayes, 2013). We did so 4 times. First, to test the moderation role of age in the association between the symbolic comparison and the Corsi forward task. It was found that both Corsi forward task (*β* = 0.21, SE = 0.05, 95% LLCI = 0.11 ULCI = 0.30) and age (*β* = 0.05, SE = 0.01, 95% LLCI = 0.02 ULCI = 0.07) was associated with symbolic comparison. The interaction was also significant (*β* = − 0.03, SE = 0.01, 95% LLCI = − 0.0026 ULCI = − 0.033), indicating that the associations between symbolic comparisons and Corsi forward task decreases with age.

Second, to test the correlation between the Corsi forward task and the non-symbolic comparison in each age, we used moderation analysis. It was found that both Corsi forward task (*β* = 0.51, SE = 0.05, 95% LLCI = 0.41 ULCI = 0.61) and age (*β* = 0.10, SE = 0.01, 95% LLCI = 0.07 ULCI = 0.12) was associated with non-symbolic comparison, and that the interaction was also significant (*β* = − 0.04, SE = 0.01, 95% LLCI = − 0.06 ULCI = − 0.03), indicating that the associations between Corsi forward and non-symbolic comparison decrease with age.

Third, we used moderation analysis, to test the moderation role of age in the association between the symbolic comparison and the Corsi backward task. It was found that both Corsi backward task (*β* = 0.19, SE = 0.05, 95% LLCI = 0.09 ULCI = 0.29) and age (*β* = 0.05, SE = 0.01, 95% LLCI = 0.03 ULCI = 0.07) was associated with symbolic comparison, and that the interaction was also significant (*β* = − 0.03, SE = 0.01, 95% LLCI = − 0.04 ULCI = − 0.01), reflecting that the effect of Corsi backward on symbolic comparison decreases with age.

**Fig. 3:**
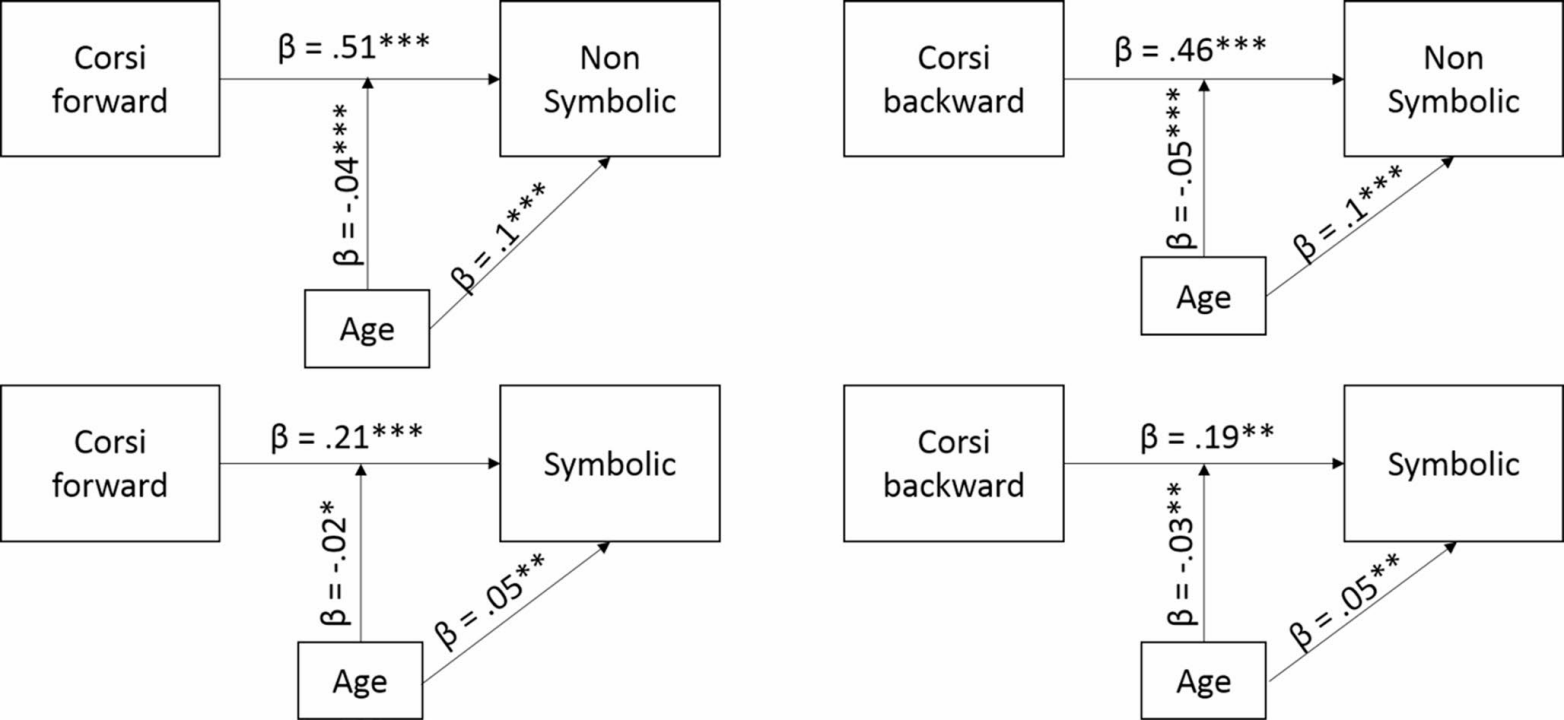
The 4 moderation analyses (model 4, one moderation in the PROCESS software). On the top row non-symbolic comparison as the dependent variable and on the bottom row symbolic comparison as the dependent variable. On the left-hand side Corsi forward as the independent variable, on the right-hand side the Corsi backwards as the independent variable. In all four models, age had both a direct positive effect and a moderation effect on numerical processing. The moderation effects were negative, indicating that the effect of age on the relations between the dependent and the independent variables decreases with age

Forth, we used moderation analysis to test the moderating role of age in the association between non-symbolic comparison and the Corsi backward task. It was found that both Corsi backward task (*β* = 0.46, SE = 0.05, 95% LLCI = 0.36 ULCI = 0.56) and age (*β* = 0.10, SE = 0.01, 95% LLCI = 0.08 ULCI = 0.12) was associated with non-symbolic comparison, and that the interaction was also significant (*β* = − 0.05, SE = 0.01, 95% LLCI = − 0.06 ULCI = − 0.03), indicating that the association between Corsi backward task and non-symbolic comparison decreases with age. See Fig. 3.

### Path analysis

Finally, in order to understand the mutual effects of all the variables in a unified model, we used the AMOS statistical software (Analysis of a Moment Structures) to create a path analysis. AMOS is an added SPSS module, and it is designed especially for Structural Equation Modeling, path analysis, and confirmatory factor analysis (Mustafa et al., 2020).

**Fig. 4:**
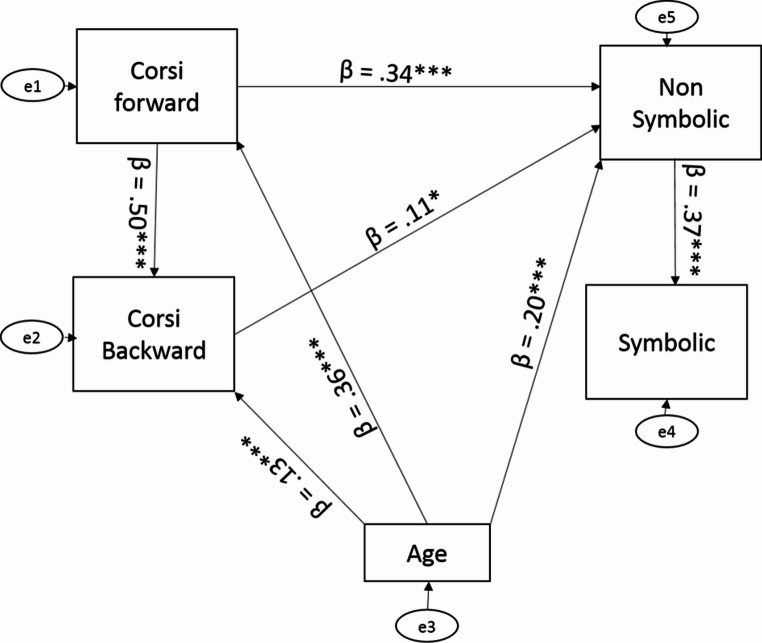
To understand the effects of all the variables in a single model, we used the path analysis. Model 1 was designed under a number of theoretical assumptions. First, we assumed that spatial abilities would influence numerical comparisons. Second, we assumed that the non-symbolic comparison would influence symbolic comparison and that Corsi forward task would influence Corsi backward task. Last, we assumed that age would directly affect all the variables, except for symbolic comparison. We found that Corsi forward task significantly associated with the non-symbolic task, but not the symbolic task. Similarly, Corsi backward task significantly associated with the non-symbolic task, but not the symbolic task. The Corsi forward task significantly associated with the Corsi backward task. Similarly, the non-symbolic task associated with the symbolic task

Model 1 of the path analysis was designed under a number of theoretical assumptions. First, we assumed that visuospatial short-term and WM abilities would influence numerical comparisons. Second, we assumed that the non-symbolic comparison would influence symbolic comparison and that Corsi forward task would influence Corsi backward task. Last, we assumed that age would directly affect all the variables, except for symbolic comparison. We found that Corsi forward task significantly predicted the non-symbolic task (*β* = 0.34, *p* <.001), but not the symbolic task (*β* = 0.07, *p* =.37). Similarly, Corsi backward task significantly predicted the non-symbolic task (*β* = 0.11, *p* <.01), but not the symbolic task (*β* = −0.05, *p* =.56.). The Corsi forward task was significantly associated with the Corsi backward task (*β* = 0.50, *p* <.01). Similarly, the non-symbolic task was associated with the symbolic task (*β* = 0.37, *p* <.01). Age was significantly associated with the Corsi forward task (*β* = 0.36, *p* <.01), the Corsi backward task (β = 0.13, *p* <.01) and the non-symbolic task (*β* = 0.20, *p* <.001). The model fit was $\:{x}^{2}\:\left(1\right)=1.08,\:p=\:.30,\:\frac{{x}^{2}}{df}=1.08,\:CFI=1,\:RMSEA=\:.01$. Then, we omitted the non-significant relationships (i.e., Corsi forward and backward and symbolic processing) and the model fit improved $\:{x}^{2}\:\left(3\right)=3.17,\:p=\:.36,\:\frac{{x}^{2}}{df}=1.06\:,\:CFI=\:1,\:RMSEA=\:.01$. See Fig. 4. Path analysis requires theoretical assumptions, such as: (1) spatial abilities influencing numerical comparisons, (2) non-symbolic comparison influencing symbolic comparison, and (3) performance on the Corsi forward task influencing performance on the Corsi backward task. In contrast, network analysis does not rely on theoretical assumptions and is therefore more data-driven. Accordingly, network analysis was employed to complement the path analysis, providing additional support for the findings obtained through the path models. Consistent with this approach, the network analysis conducted without a priori assumptions yielded similar results (see Supplementary Material).

## Discussion

Spatial abilities, and especially short-term memory and WM, are essential for building mathematical abilities (Cornu et al., 2018; Geer et al., 2019; Lauer & Lourenco, 2016). A recent study, with a similar age range, discovered that spatial skills directly and strongly affect numerical comparison, and specifically, spatial short-term memory is the strongest predictor of all the spatial abilities measured (Ashkenazi & Adi, 2023). Accordingly, the main aim of the current study was to look at the developmental paths of the association between spatial abilities and numerical abilities. Previous studies indicated that both spatial WM and short-term memory are associated with numerical performance(Alloway & Alloway, 2010; Bull et al., 2008; Bull & Lee, 2014; Cragg & Gilmore, 2014; De Smedt et al., 2009; DeStefano & LeFevre, 2004; Friso-van den Bos et al., 2013; Passolunghi & Costa, 2019; Peng et al., 2012; Raghubar et al., 2010). However, the strength of the associations is dependent on the numerical task used and on the age of the participants. In order to further deepen our understanding, the present study tested the role of spatial short-term memory and WM in numerical processing in a very large sample of children (*n* = 541; ages 2.5 to 7.5). We tested basic numerical tasks: (1) symbolic comparison, (2) non-symbolic comparison and visuospatial tasks: (1) short-term memory (tested by the Corsi forward task) and (2) WM (tested by the Corsi backwards task). Initially, we looked at the effect of development on the performance in the spatial tasks. As expected, both short-term memory and WM span increased with age. These results are in line with previous studies that showed development in WM domains (van der Ven et al., 2013).

Next, we looked at the relations between the different tasks to better understand the role of spatial abilities in mathematical abilities. We discovered a positive relation between both non-symbolic and symbolic comparison and spatial abilities. Finally, we examined the moderation effect of age on the relationship between numerical tasks and spatial abilities and looked at all the variables in one unified model. The associations between spatial abilities and numerical abilities were stronger in younger children than in older children. Namely, in all the tasks, there was significant modulation of age on the relationship between spatial and numerical tasks. However, there were also differences between tasks. The associations between spatial abilities and non-symbolic comparisons where stronger than the associations between spatial abilities and symbolic comparisons. In fact, when combining all the variables, it appeared that spatial short-term memory is the strongest predictor of non-symbolic comparison. Spatial WM independently contributes significantly to the explanation of variance of non-symbolic numerical comparison. However, there was no direct effect of spatial short-term memory or WM on symbolic comparison, only indirect effect via non-symbolic comparison. Additionally, symbolic comparison was strongly influenced by non-symbolic comparison. Finally, in the unified model, age effected both spatial short-term memory and WM and also non-symbolic comparison. However, age did not show direct effect on symbolic comparison. These results demonstrate that there is no direct link between symbolic comparison and spatial abilities.

### The role of spatial short-term memory and WM in non-symbolic comparison

It has been found, throughout various studies, that both individuals with mathematical learning disabilities and those who are typically developing, rely heavily on their spatial abilities to perform well in numerical and mathematical tasks (Ashkenazi & Adi, 2023; Ashkenazi et al., 2013; Ashkenazi & Silverman, 2017; Danan & Ashkenazi, 2022; Silverman & Ashkenazi, 2022). Specifically, three recent meta-analyses explore the relations between spatial abilities and mathematical abilities in children (Allen et al., 2019b; Friso-van den Bos et al., 2013; Zhang et al., 2023). Allen et al. (2019b) discovered positive relations between spatial short-term memory and WM and mathematical abilities in children (*r* =.4). The correlations were stable with age (see also (Zhang et al., 2023) for similar results). Friso-van den Bos et al. (2013) looked at children in primary school and found correlations between spatial short-term memory and mathematical abilities. The correlations were highest using the Corsi forward task (the same task as in the present study) (β = 0.43) compared to all the other spatial short-term memory tasks used. Moreover, the correlations were similar when different mathematical tasks were used, including word problems, counting and concepts. Contrary to Allen et al. (2019a), age did not harm that association. With respect to spatial WM, they discovered a correlation with mathematical abilities (*r* =.34), and this association increased with age. Our results regarding short-term memory were consistent with Friso-van den Bos et al. (2013), with the strength of correlation decreasing with age. However, regarding the association between spatial WM and numerical abilities, we discovered opposite relations compared to Friso-van den Bos et al. (2013). One explanation for this inconsistency between our results and the results of others (Allen et al., 2019b; Friso-van den Bos et al., 2013; Zhang et al., 2023) could be the difference in the mathematical tasks used. It has been reported that the associations between spatial WM and mathematical abilities is stronger in complex mathematical tasks than in simple mathematical tasks (Friso-van den Bos et al., 2013). In the current study, a very basic numerical task was used (numerical comparison). For younger children, numerical comparison is not automatic and requires WM (Kroesbergen et al., 2014). However, for school-age children, quantity comparison becomes easier, potentially resulting in a smaller correlation with WM. When a task becomes easier, it requires fewer working memory resources. Hence, the effect of age on the relation between spatial WM and mathematical abilities is dependent on the specific task used. Please note, however, that a recent study examined the role of visuospatial WM in adults, using a paradigm similar to that of the present study (Corsi). The study, conducted in the context of numerical comparison (Xenidou-Dervou et al., 2025), found that visuospatial WM is required for both symbolic and non-symbolic comparison.

In line with the view that connects spatial WM to more complex tasks in mathematics, in a study testing the role of spatial short-term memory and WM in explaining individual differences in mathematics, subtraction had a higher association with spatial WM compared to multiplication. Additionally, the associations were higher in younger children than in older children, only in subtraction. In multiplication, the associations were similar in all ages (van der Ven et al., 2013).

One of the main explanations for the decrease in the correlation between spatial abilities and numerical abilities with age, may be the different strategies used to preform numerical tasks in preschool children, that had not yet acquired the exact verbal representation of quantities (Rasmussen & Bisanz, 2005). Children are born with an intuitive ability to manipulate and understand approximate magnitudes, that is primarily based on a spatial mental representation (Feigenson & Halberda, 2004; Halberda & Feigenson, 2008; Halberda et al., 2008, 2012). Later, with formal education, a verbally mediated exact numerical representation is acquired. Hence, it has been suggested that, before the acquisition of verbal representation of numbers, younger children commonly rely on a mental model that is based on spatial representation or on spatial strategies (Rasmussen & Bisanz, 2005). As formal education progresses and exact symbolic representation is acquired, participants tend to shift to more verbal strategies (Shalev & Gross-Tsur, 2001). Please note that, in some of the countries, the mathematical curriculum encourages more verbal strategies over spatial ones (Holmes & Adams, 2006).

For example, in the current non-symbolic comparison, either spatial or verbal strategies can be applied to perform this task. The One-To-One matching method is primarily a visuospatial strategy, that can be applied before the acquisition of verbal strategies (Izard et al., 2014). In that strategy, participants are paring items between the two matched sets, until there are no more items to pair in one of the sets. Alternatively, a child can use a verbal strategy where every quantity is first verbally counted, and then the child determines which is the larger set, according to the total number of items in each set. Hence, spatial abilities are more important in younger children, before the beginning of formal mathematical education, resulting in decreased association between spatial abilities and numerical abilities with age (Fanari et al., 2019).

### The relationship between symbolic and non-symbolic processing - the role of spatial abilities

Symbolic representation of quantities (5 = *****) is culturally mediated. With explicit training, the exact mapping between symbol and quantity is acquired. However, the understanding of approximate quantities, by spatially mediated representation, is innate and based on defined neurological circuits (Ashkenazi et al., 2013; Dehaene, 2009; Dehaene et al., 1999, 2003; Halberda & Feigenson, 2008). That being said, it is not clear whether the culturally symbolic exact representation of numbers is built upon the innate approximate representation (Halberda & Feigenson, 2008; Halberda et al., 2008) or developed separately (Carey, 2001, 2004; Noel & Rousselle, 2011). Noel and Rousselle (2011) suggest that the symbolic and non-symbolic systems are largely independent, and that symbolic comparison, but not non-symbolic comparison, mostly affects mathematical abilities later on. In order to decide which notion is more reasonable, the second goal of our study, to look at the differential role of spatial abilities in symbolic and non-symbolic numerical comparisons, proved useful.

A few aspects of our results support the notion that symbolic representations are directly mapped onto non-symbolic representations. (1) The correlation between spatial abilities and non-symbolic comparisons was stronger than the correlation between symbolic comparisons and spatial abilities. Due to the more complex spatial presentation of non-symbolic numerical representations, compared to symbolic representations, this result is in line with the fact that comparing non-symbolic quantities requires greater spatial abilities compared to symbolic comparisons. Interestingly, (2) when including all variables in one unified model, there was no direct effect of spatial abilities on symbolic comparison; all the effects were indirect, via non-symbolic comparison. All of these results suggest that symbolic representations are directly mapped upon non-symbolic representations.

### Limitations

The present study examined the role of spatial abilities in numerical processing by using a cross sectional approach. We asked whether the association between spatial abilities and numerical processing changes with development. The longitudinal approach is a better way to examine this question. However, due to the very large sample size and wide range of ages, we believe that the current data set provides us with valuable information regarding this question. It would be important to use the same task designs in future studies, testing the same participants at multiple time points.

Moreover, analyses were conducted only on participants with complete data for all tasks, reducing the sample to 541 children out of the initial 4,127. This procedure may introduce selection bias. For example, it is possible that only children with higher mathematical abilities or better sustained attention completed all tasks. This bias may be particularly pronounced in younger age groups, especially among the three-year-olds, thereby limiting the generalizability of the findings. Moreover, because participation was voluntary and the tool was administered online, it was not possible to determine the participants’ nationality with certainty. In addition, the study took place in a non-controlled environment, meaning that children completed the tasks at different times and sessions according to their own choice. These limitations may affect the generalizability of the findings.

Finally, it is important to note that the application did not allow for control over the context in which children completed the tasks. Children participated in various environments and were able to complete the tasks across multiple sessions, depending on their individual needs. Additionally, due to technical constraints, the app assessed only visuospatial short-term memory and working memory, without including verbal short-term or working memory. Nevertheless, parts of the discussion are based on the assumption of an increased reliance on visuospatial short-term memory and working memory with age. Accordingly, it will be important in future studies to test the role of visuospatial and verbal WM throughout development.

## Conclusion

One of the main goals that guided the present study was to understand the role of spatial abilities in numerical processing and the modulation effect of age on these relations. As expected, we found that the quantities comparison tasks are directly associated, primarily, with spatial short-term memory and, to a lesser degree, with spatial WM. Because these two associations decrease with age, we argue that younger children are using a spatial strategy more than verbally mediated strategies during numerical comparison tasks. The associations were stronger between non-symbolic comparison and spatial abilities compared to symbolic comparison and spatial abilities. In fact, when including symbolic and non-symbolic comparisons in one model, no direct link between symbolic comparison and spatial abilities is found. Similarly, in the unified model, age affected non-symbolic comparison abilities but not symbolic comparison abilities. These results demonstrate that symbolic representation is built upon non-symbolic representation.

## Supplementary information

Below is the link to the electronic supplementary material.

ESM 1 (DOCX 126 KB)

## Data Availability

The data will be given upon request from the first author.
