# Supplementary material for: Developmental paths of the associations between visuospatial working memory and numerical processing
Source: Psychol Res. 2026 Feb 7;90(1):23. doi: 10.1007/s00426-026-02240-6 (PMC12881172; doi:10.1007/s00426-026-02240-6)
Supplement: Supplementary file 1 — (DOCX 126 KB) [file 426_2026_2240_MOESM1_ESM.docx]

Supplementary material

To determine the relationships between all the spatial and numerical dimensions without a-priori assumptions, we performed a network analysis. Using JASP software, we calculated a sparse Gaussian graphical model with the graphical lasso ([Zhao & Iyengar, 2012](#_ENREF_7)). This allowed us to look at the complex relations between numerical and spatial abilities from a mutualism perspective. Therefore, the network graph can represent the different relationships and mediation pathways, which can serve as a valuable hypothesis for the potential structure of particular nodes ([Hevey, 2018](#_ENREF_5)).

Five nodes formed 8 significant connections. The network analysis was based on an EBICglasso. Weights matrix discovered that **age** was related to 1) non-symbolic task (0.184), 2) Corsi forward task (0.153) and 3) Corsi backward task(0.114). **Corsi forward** task was related to 1) Corsi backward task (0.420) and 2) non-symbolic task (0.277). As mentioned, **Corsi backwards** task was related to 1) Corsi forward task and 2) non-symbolic task (0.102). **Symbolic task** was related only to non-symbolic task (0.285).

Figure S1. Network analysis lines represent associations between two nodes in the network. The thickness of the line represents the strength of the association, blue lines represent positive associations.
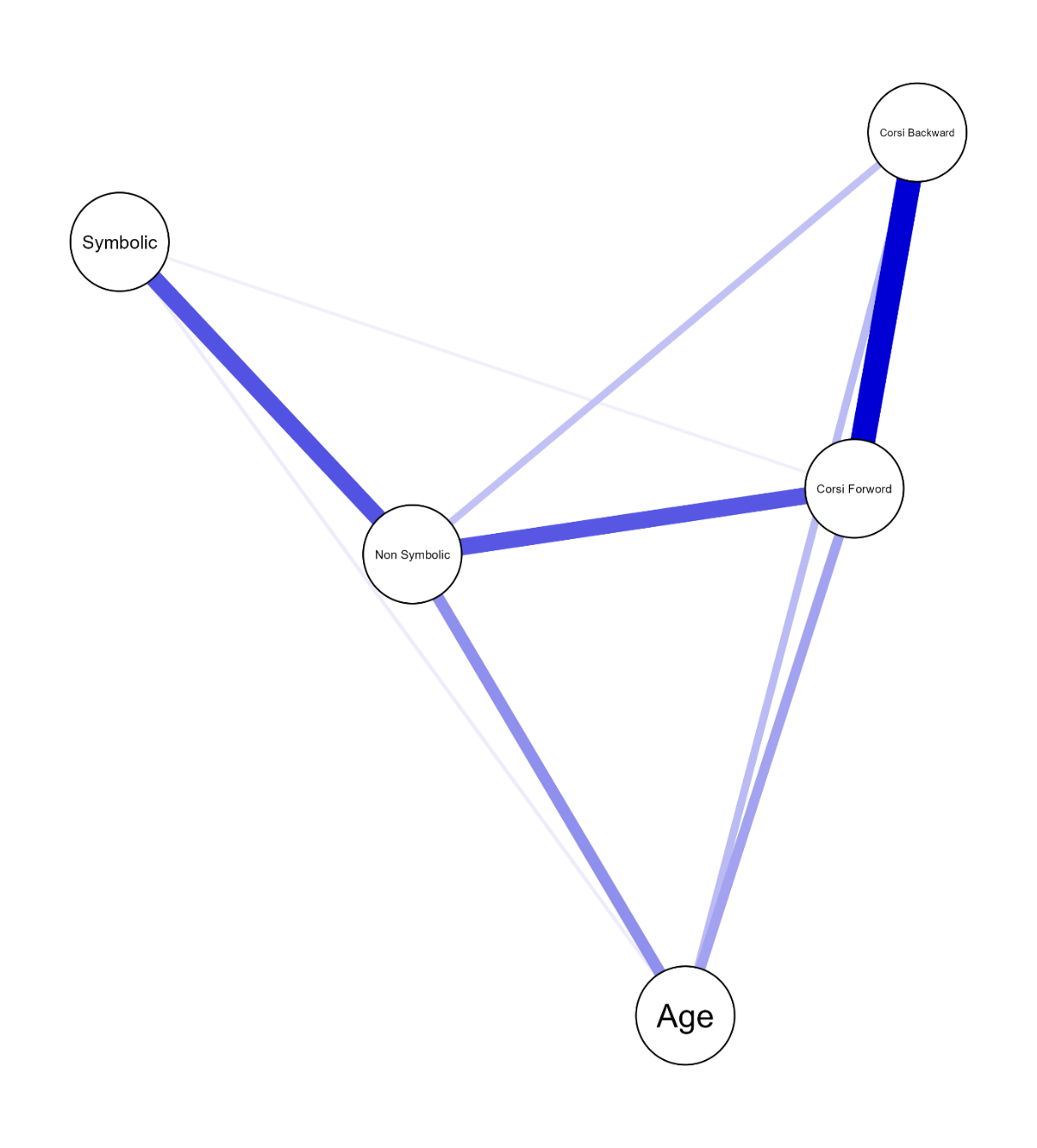


Refrences for the SM

Hevey, D. (2018). Network analysis: a brief overview and tutorial. *Health psychology and behavioral medicine*, *6*(1), 301-328.

Zhao, S., & Iyengar, R. (2012). Systems pharmacology: network analysis to identify multiscale mechanisms of drug action. *Annual review of pharmacology and toxicology*, *52*(1), 505-521.
